# Editorial: Applications of fast breeding technologies in crop improvement and functional genomics study

**DOI:** 10.3389/fpls.2024.1460642

**Published:** 2024-07-19

**Authors:** Xingguo Ye, Fangpu Han

**Affiliations:** ^1^ Institute of Crop Sciences, Chinese Academy of Agricultural Sciences, Beijing, China; ^2^ Institute of Genetics and Developmental Biology, Chinese Academy of Sciences, Beijing, China

**Keywords:** crop, fast breeding, haploid induction, molecular marker, gene editing

New crop varieties are increasingly crucial to agriculture production, and their development depends on the exploration and application of key germplasm resources, often referred to as seed chips. Conventional breeding strategies are time-consuming, but recent advances in fast breeding technologies have significantly accelerated the process of crop trait modification, gene mapping, and functional genomics studies. These technologies include molecular selection, molecular mapping of genes, haploid induction, and genome editing mediated by CRISPR/Cas9 (**Figure 1**) (Gao, 2021). CRISPR/Cas9 has been applied to rapidly modify important crop traits such as disease resistance, quality, maturity, grain weight, sterility, and pre-harvest sprouting tolerance (Li et al., 2024). Particularly, new haploid inducer lines have been created in maize, rice, *Arabidopsis*, wheat, alfalfa, foxtail millet, tomato, *Brassica oleracea*, and soybean by editing *MTL* (*PLA1*/*NLD*), *DMP*, and *PLD3* genes *via* CRISPR/Cas9 to produce haploid grains directly (Qi et al., 2023; Tang et al., 2023; Qu et al., 2024). Additionally, new types of molecular markers have been developed and used to trace agronomically important traits in breeding programs and locate target genes on chromosomes for gene cloning alongside generally employed makers such as simple sequence repeat (SSR), single-nucleotide polymorphism (SNP), and expressed sequence tags (EST) (Rasheed et al., 2017; Hao et al., 2020).

**Figure 1:**
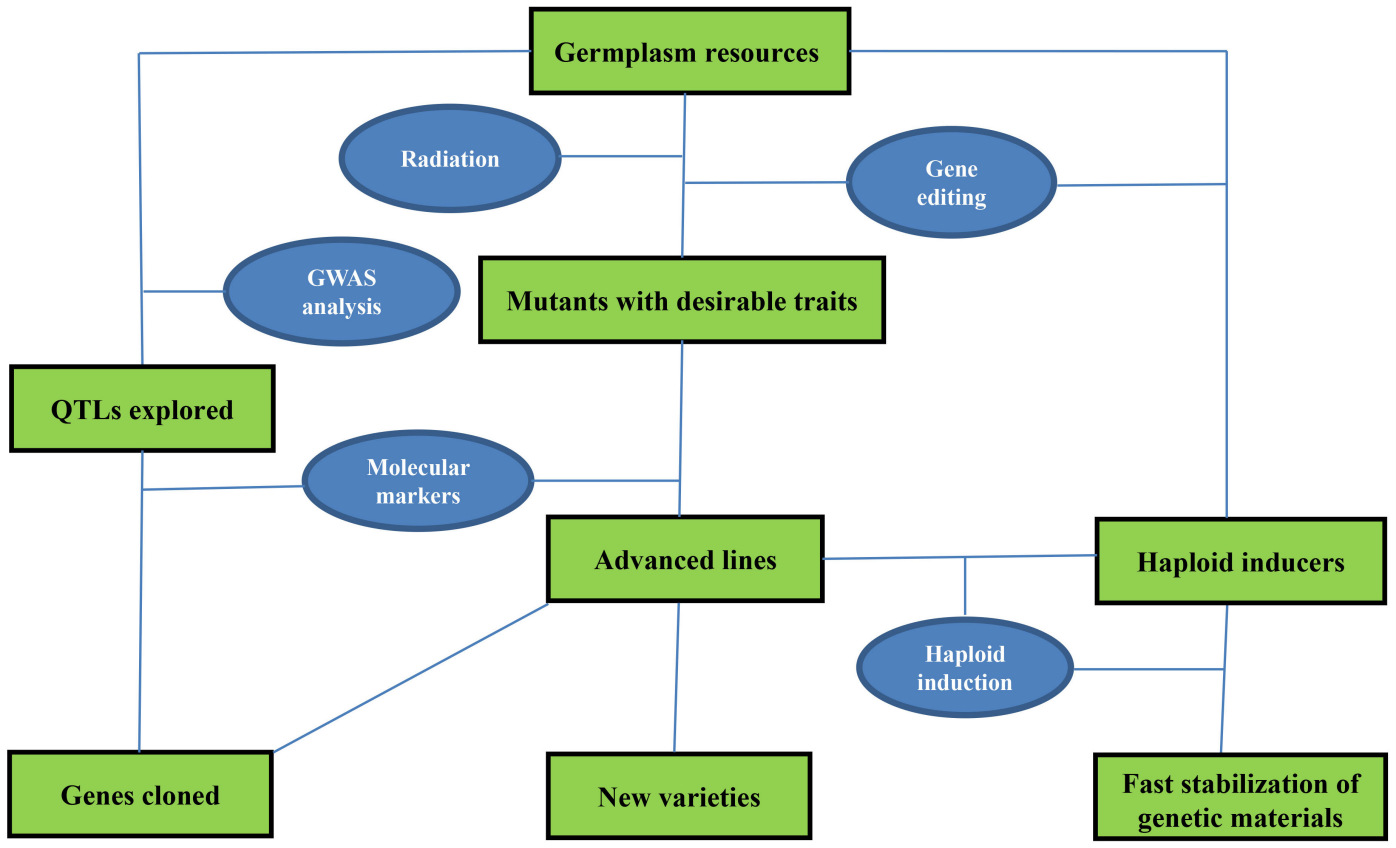
Fast breeding techniques and their application in genetic improvement and functional dissection of target genes in crops.

Using these techniques, the period for breeding new crop varieties and germplasms can be greatly shortened. Therefore, we organized this Research Topic to collectively report the achievements in the applications of fast breeding technologies in genetic improvement and functional genomic studies in various plants. Since the initiation of this Research Topic in June 2023, we have received 39 manuscripts on the development, application, and summarization of fast breeding technologies in plants. Finally, 15 papers have been published in this Research Topic after careful evaluation.

Melon and potato are important fruit and vegetable crops worldwide, and their genetic improvement and resource expansion can be efficiently promoted by the exploration of quantitative trait loci (QTL) and molecular markers. In the melon paper, the resistance gene to downy mildew (DM) was mapped using linkage map and QTL-seq analysis, conducted with two F_2_ populations that included a DM-resistant accession. A high-density genetic map was generated, and a major QTL, *DM9.1*, was consistently identified. This QTL was further validated through QTL-seq analyses and then precisely mapped to the chromosome using Kompetitive allele-specific PCR (KASP) assays. A KASP marker for *DM9.1* was also developed. In the two potato papers for the identification of disease resistance and yield traits associated with QTL, some significant or common QTL for all five traits (late blight, *Verticillium* wilt, early blight resistances, vine size, and maturity) or isolated instances of the traits were detected on chromosomes 1, 2, 3, 5, and 10. Additionally, a few major or significant QTL were detected on chromosomes 4 and 10 for tuber shape, on chromosome 2 for width–depth ratio, on chromosome 5 for tuber weight, and on chromosome 3 for specific gravity.

Wheat is a globally important food crop. It is necessary to improve wheat using the marker-assisted selection strategy (Hao et al., 2020). In the four wheat papers, firstly, authors found a major QTL for grain hardness, *QGh.cib-7D*, which is widely present in wheat, by identifying a recombinant inbred line (RIL) population for quality-related traits by QTL. The most potential candidate genes at *QGh.cib-7D* locus were initially deduced. Secondly, authors identified the interval of *QPh-1B* including an EST-SSR marker, *swes1079*, for plant height. According to the sequence of *swes1079*, they cloned the *TaOSCA1.4* gene and found that the gene was significantly different between the two haplotypes of *TaOSCA1.4-1B* by detecting a natural population using a developed a CAPS (cleaved amplification polymorphism sequence-tagged sites) marker. Thirdly, based on the fact that an RIL population exhibited segregation in resistance to powdery mildew and stripe rust, a 16K genotyping by target sequencing (GBTS) SNP array-based genetic linkage map was used to dissect the QTL for disease resistance. Eleven QTL were identified for adult-plant resistance against powdery mildew and stripe rust. A KASP marker, *KASP_1B_668028290*, was developed to trace the major QTL, *QPm/Yr.caas.1B*, for resistance to both powdery mildew and stripe rust. New lines pyramiding six loci of *PmQ*, *Yr041133*, *QPm/Yr.caas-1B*, *QPm.caas-2B.1*, *QYr.caas-3B*, and *QPm.caas-6B* were developed, displaying effective resistance against powdery mildew and stripe rust. Fourthly, a platform was established for the systematic evaluation of *Aegilops tauschii* traits in the background of wheat. This platform was designed to identify and mine useful QTL and genes by widening genetic diversity. A multiple synthetic derivative population, which included 43 *Ae. tauschii* accessions, was utilized. Among these accessions, nine were found to exhibit the seed dormancy trait, with KU-2039 displaying the highest level of seed dormancy. QTL mapping using backcross inbred lines derived from KU-2039 revealed a major QTL, *Qsd.alrc.5D*, associated with dormancy.

Crop mutants can be produced by irradiation and gene editing (Gao, 2021). In the rice paper, a total of 129 rice mutants with distinct phenotypic variations, including changes in agronomic traits, were screened from more than 10,000 M_2_ plants by irradiating the mature dry seeds with an optimized dosage of carbon ion beams. Whole-genome sequencing on M_2_ individuals revealed that the mutation rate peaked at this dosage. Nearly half of the mutants possessed stable inheritance in M_3_ generation, and three lines in the M_4_ generation showed an increase in yield. In the tomato paper, authors edited the tomato *SlHyPRP1* and *SlDEA1* genes, which were recently identified as being responsive to different abiotic (drought and salinity) and biotic stress (bacterial leaf spot and bacterial wilt) conditions. The mutants showed higher levels of chlorophyll and proline contents under abiotic stress conditions and less reactive oxygen species accumulation and cell death of leaves and roots under biotic stress compared to the wild type. The combined loss-of-function of *SlHyPRP1* and *SlDEA1* was essential for imparting significant multi-stress tolerance in tomato.

Doubled haploid (DH) production through *in vivo* maternal haploid induction provides an effective way to generate homozygous genetic and breeding materials over a short period, which is widely applied in maize breeding programs (Ishii et al., 2016; Qu et al., 2024). In the maize mini review on haploid identification, authors reviewed various methods for haploid identification by different approaches in maize including innate differences in haploids and diploids, biomarkers integrated in haploid inducers, and automated seed sorting. The phenotypic differentiation, genetic basis, advantages, and limitations in each biomarker system were highlighted. Automated high-throughput haploid sorting with high accuracy and less workforce or budget in a time-saving manner would be promising in the near future.

In the durum wheat paper on the development of a haploid inducer, authors produced three types of homozygous *TtMTL* gene-edited mutants (*mtl-a*, *mtl-b*, and *mtl-ab*) by CRISPR/Cas9. Among these, the mutants *mtl-a* and *mtl-ab* showed a decreased seed-setting rate and some embryo-abortion grains and triggered haploids with an available rate in the cross- and self-pollinated progenies. Furthermore, authors pyramided *Ttmtl*-edited mutations and embryo-specific expressed anthocyanin markers and developed a homozygous durum haploid inducer with purple embryo (DHIPE) by co-transformation of the two vectors *pCRISPR/Cas9-MTL* and *pBD68-*(*ZmR+ZmC1*). Using DHIPE as the male parent to be crossed with other wheat materials, the grains with white embryos were identified as haploid, and the grains with purple embryos were diploid.

In the rapeseed paper on the possible mechanism of maternal doubled haploid formation, a rapeseed inducer containing an exogenous gene *cp4-EPSPS* as a marker, which can induce maternal doubled haploids when used as a pollen donor, was employed as paternal material to cross with maternal materials. At the early stage after pollination, *cp4-EPSPS* was detected in the embryos; at the middle stage, *cp4-EPSPS* was present in the majority of embryos, but it was lost at the late stage. The expression of *cp4-EPSPS* could be detected in the early development period of the embryos, but it could not be detected in the surviving embryos in the late development stage. Meanwhile, double haploids in the induced offspring were confirmed through SNP detection. The results indicated that the chromosomes of the inducer were eliminated during embryonic development, and the maternal haploid chromosomes were synchronously doubled in the embryo.

Virus-induced gene silencing (VIGS), genome-wide association study (GWAS), and machine learning methods also contribute to the exploration and dissection of gene resources in plants. In the paper on the establishment of a simple and efficient VIGS method, a root wounding–immersion method was developed, in which plant roots were partly cut and immersed in a *Tobacco rattle virus* (TRV)1:TRV2 mixed solution. The authors further optimized the procedure in tobacco and silenced phytoene desaturase gene *PDS* in tobacco, tomato, pepper, eggplant, and *Arabidopsis* and observed the movement of green fluorescent protein (GFP) from roots to stems and leaves. In addition, two disease-resistance genes, *SITL5* and *SITL6*, in tomato were silenced by this method, and the generated plants showed a decreased disease resistance. In the chickpea paper, a genome-wide association studies (GWAS) strategy was conducted to unravel genetics on heat tolerance. The authors extensively evaluated chickpea genotypes for the traits associated with yield and heat stress tolerance, finding significant variations. Furthermore, they identified linked marker–trait associations (MTAs) to yield-related traits by performing SNP genotyping using genotyping-by-sequencing for GWAS analysis. A few MTAs displayed pleiotropic effects, and the majority of the SNPs were located at or in proximity to gene-coding regions, with the candidate genes situated near these MTAs. The harvest index trait was associated with marker Ca 3:37444451, encoding aspartic proteinase, which contributes to heat stress tolerance. In the machine learning paper, authors evaluated four types of algorithms for genomic predictions of clonal performance for disease resistance in sugarcane. The results indicated that hybrid Bayesian-machine learning methods with attention networks provided the lowest variation in accuracy of genomic prediction of clonal performance across validation folds, which may be an additional criteria worth considering in practical breeding programs.

In summary, the papers published in this Research Topic described interesting cases on new gene exploration and function dissection, breeding material and mutant creation by radiation and gene editing, and molecular marker development and their applications in assisted selection and gene mapping for important traits in various crops or plants. The resulting information will accelerate the development of new germplasms and varieties, as well as the cloning of new genes in different crops, which will be of critical importance for humanity in the coming years.

## Author contributions

XY: Writing – original draft, Writing – review & editing. FH: Writing – review & editing.
